# Detection of environmental nanoplastics via surface-enhanced Raman spectroscopy using high-density, ring-shaped nanogap arrays

**DOI:** 10.3389/fbioe.2023.1242797

**Published:** 2023-10-24

**Authors:** Sihai Luo, Junjie Zhang, John C. de Mello

**Affiliations:** Department of Chemistry, Norwegian University of Science and Technology (NTNU), Trondheim, Norway

**Keywords:** nanoplastics, microplastics, surface-enhanced Raman spectroscopy, lithography, plasmonics

## Abstract

Micro- and nano-plastics (MNPs) are global contaminants of growing concern to the ecosystem and human health. In-the-field detection and identification of environmental micro- and nano-plastics (e-MNPs) is critical for monitoring the spread and effects of e-MNPs but is challenging due to the dearth of suitable analytical techniques, especially in the sub-micron size range. Here we show that thin gold films patterned with a dense, hexagonal array of ring-shaped nanogaps (RSNs) can be used as active substrates for the sensitive detection of micro- and nano-plastics by surface-enhanced Raman spectroscopy (SERS), requiring only small sample volumes and no significant sample preparation. By drop-casting 0.2-μL aqueous test samples onto the SERS substrates, 50-nm polystyrene (PS) nanoparticles could be determined via Raman spectroscopy at concentrations down to 1 μg/mL. The substrates were successfully applied to the detection and identification of ∼100-nm polypropylene e-MNPs in filtered drinking water and ∼100-nm polyethylene terephthalate (PET) e-MNPs in filtered wash-water from a freshly cleaned PET-based infant feeding bottle.

## 1 Introduction

Micro- and nano-plastics (MNPs) have received substantial attention in recent years as emerging contaminants of significant concern due to their increasing prevalence in the environment and the threat they pose to ecosystems, aquatic organisms and human health (Rillig, 2020; Rochman and Hoellein, 2020; Revell et al., 2021; Su et al., 2021; Vethaak and Legler, 2021). Nanoplastics (NPs) of diameter *d* << 1 µm are of particular concern as their small size means they tend to be more readily distributed in living systems than microplastics (*d* > 1 µm) (Wright and Kelly, 2017; Xu et al., 2020; Gigault et al., 2021). The high specific surface-area of NPs increases their ability to absorb pollutants, while their small size allows them to easily enter cells, which can lead to bioaccumulation of plastics and absorbed pollutants, adversely affecting metabolism, growth, reproduction, feeding, oxidative balance, antioxidative capacity, and neurological and immunological function (Gigault et al., 2021; Song et al., 2021). There is increasing evidence that infection rates due to microparasites are strongly affected by the presence of environmental nanoplastics (e-NPs), either increasing or decreasing infection rates depending on the type of parasite (Manzi et al., 2023).

The prevalence of environmental nanoplastics (e-NPs) has been less widely investigated than that of environmental microplastics (e-MPs) due in part to the lack of effective in-the-field methods for detecting and identifying sub-micron sized plastic particulates (Ivleva et al., 2017; Alimi et al., 2018; Mitrano et al., 2019; Sander et al., 2019). Various techniques have been reported for detecting NPs, including infrared absorption spectroscopy (IRAS) (Tagg et al., 2015; Ivleva et al., 2017; Cabernard et al., 2018; Su et al., 2021), Raman spectroscopy (RS) (Ivleva et al., 2017; Araujo et al., 2018; Sobhani et al., 2019; Wright et al., 2019), pyrolysis gas chromatography/mass spectrometry (GC-MS) (Ter Halle et al., 2017; Laurentie et al., 2018; Dierkes et al., 2019), liquid chromatography/mass spectrometry (LC-MS) (Wang et al., 2017; Peng et al., 2020), electron microscopy, (Wang et al., 2017; Ivleva et al., 2017; Pivokonsky et al., 2018; Mitrano et al., 2019), fluorescence microscopy (Cole and Galloway, 2015; Erni-Cassola et al., 2017; Maes et al., 2017), and dynamic light scattering (DLS) (Gigault et al., 2016).

Methods involving chromatographic separation followed by mass spectrometric detection (in particular LC-MS and GC-MS) are particularly widely used for analysing environmental samples due to the high sensitivity they can provide down to the ng/L level and beyond. However, the need for extensive sample pre-treatment, and the complexity, high cost and limited portability of the associated instrumentation preclude their use for most field studies (Ong et al., 2020). Electron microscopy is similarly ill-suited to in-the-field deployment, while fluorescence spectroscopy and DLS lack specificity.

Of the aforementioned techniques for e-NP detection, infrared and Raman spectroscopy are the easiest to apply at remote locations due to their simplicity and ease of miniaturisation. However, conventional IRAS and RS methods (involving the direct interaction of incident light with the target molecules) have insufficient sensitivity for the detection of e-NPs at trace concentrations. Raman spectroscopy in particular suffers from very poor sensitivity as it involves *inelastic* scattering of incident photons by the target analyte, a process that occurs with low efficiency (Xie et al., 2022).

Surface-enhanced infrared absorption spectroscopy (SEIRAS) and surface-enhanced Raman spectroscopy (SERS) overcome the weak sensitivity of conventional IRAS and RS methods by using a plasmonically active substrate to enhance the interaction of the incident light with the target molecules. In both cases, the substrate acts as a receiving antenna that improves the efficiency with which incident light is coupled to the target molecules while, in the case of SERS, it also acts as a transmitting antenna that enhances the efficiency with which the inelastically scattered light is re-emitted into the far-field (Chen et al., 2015; Luo et al., 2021).

SERS has attracted particular interest as a method for detecting environmental chemical hazards due to its high sensitivity, ease of implementation, miniaturisability, fast analysis speeds, and good selectivity based on the distinct Raman signature of target analytes (Cecchini et al., 2013; Fu et al., 2017; Zong et al., 2018; Nie et al., 2019). By immobilising the target molecules on a structured metallic substrate containing a high density of nanoscale features where the local electric field is greatly enhanced, Raman scattering signals can be increased by a factor of 10^8^ or more compared to a passive (SERS-inactive) substrate (Xie et al., 2022). Thus Raman spectroscopy may be transformed from a low sensitivity technique into a highly sensitive one that may even be used for single molecule detection (Langer et al., 2019).

A key advantage of SERS over SEIRAS is that it uses simple detection optics that are well-suited to in-the-field deployment–a complete SERS set-up can be as simple as a SERS-active substrate and a visible or near-infrared (NIR) light-source plus a notch or edge filter and visible/NIR spectrometer for detection (Somerville et al., 2010). SEIRAS, by contrast, requires a broadband mid-IR light-source and a (costly) mechanical interferometer for detection, and cannot usually match the exquisite sensitivity of SERS (Ong et al., 2020) Limitations of SERS for molecular detection include potential dependence of the SERS spectrum on the orientation of the deposited molecules (although this may be less of a concern for the detection of NPs), the difficulty of analysing environmental samples containing multiple Raman-active analytes, and the difficulty of achieving quantitative detection. Nonetheless, SERS is rapidly developing into a practical tool for environmental analysis, and its potential application to the detection of e-MNPs merits further investigation (Halvorson and Vikesland, 2010).

To date, there have been relatively few reports in the literature concerning the detection of NPs on SERS-active substrates. By drop-casting 5-μL aqueous samples of 400-nm (diameter) polystyrene (PS) NPs onto a SERS substrate formed from silver-coated gold nanoparticles embedded in anodized aluminium oxide arrays, Trung et al. reported successful SERS detection at concentrations down to 50 μg/mL (Trung et al., 2021). By drop-casting a mixture of ∼50-nm polystyrene NPs and silver nanoparticles onto a silicon wafer, Zhou et al. reported SERS detection at a concentration of 100 μg/mL (Zhou et al., 2021). The Ag/NP mixture was formed by combining a 50-μL NP solution with a 1-mL dispersion of Ag nanoparticles and a 50-μL solution of MgSO_4_ as a coagulant, with a SERS substrate in effect being formed as the Ag/NP solution dried on the (SERS-inactive) silicon wafer. By drop-casting 100-μL aqueous samples of 360-nm polystyrene NPs onto a commercial SERS substrate known as Klarite, Xu et al. reported successful detection at a concentration of 30 μg/mL. (Xu et al., 2020). Klarite is a highly engineered material formed from a square grid of gold-coated, lithographically etched pyramid-shaped wells in a silicon wafer, and its high reported cost of ∼US$100 for a 4 mm^2^ substrate is a barrier to its wider use in e-MNP detection (Shinki and Sarkar, 2022). By drop-casting 0.2 μL aqueous samples of 50-nm PS nanoparticles onto an array of triangular cavities in a thin gold film, Zhang et al. reported detection of polystyrene nanospheres at a concentration down to 10 μg/mL. (Zhang et al., 2023). Using a mesh of silver nanowires (AgNWs) as a SERS substrate, Yang et al. reported a detection limit of 0.1 μg/mL for 50-nm PS nanoparticles in water (Yang et al., 2022). In this case, however, the very low (i.e., very good) detection limit was largely attributable to the use of a high 1-mL sample-volume that was pumped through the AgNW mesh using a syringe pump, with the mesh acting as a trap for the passing MNP particles. Chaisrikhwun et al. reported a detection limit of 0.1 μg/mL for 100-nm polystyrene NPs in water, using sputtered gold on glass as a SERS substrate (Chaisrikhwun et al., 2023). In this approach substantial sample handling was required prior to SERS detection, with 500-μL aqueous samples first being dried and then dissolved in toluene to destroy the particulate structure of the MNPs before depositing the solvated PS chains as a thin film on the SERS substrate.

The above reports confirm the feasibility of using SERS for the sensitive detection of NPs, but considerable challenges remain. In particular there is a need for easy-to-use, low cost and preferably re-usable SERS substrates for the trace detection of NPs in the size range 50–1000 nm that can be used with small sample volumes (<< 10 μL), require minimal sample preparation, and offer high spatial uniformity in their SERS activity. In this work, we describe a simple platform for SERS detection of NPs based on thin gold films patterned with a dense, hexagonal array of ring-shaped nanogaps (RSNs). The RSN arrays may be readily fabricated over large areas of up to 50 mm^2^ using a combination of two lithographic approaches: nanosphere lithography and adhesion lithography. (Beesley et al., 2014; Luo et al., 2021; Luo et al., 2022b). We show here that, by drop-casting 0.2-μL samples of water-dispersed e-MNPs directly onto the RSN substrates, NPs as small as 50-nm may be detected at concentrations of ∼ 1 μg/mL using SERS spectroscopy.

## 2 Materials and methods

### 2.1 Materials

Borosilicate glass substrates were cleaned by oxygen plasma before use. Octadecanethiol (ODT, 98%), 10 wt% aqueous suspensions of 200-nm, 500-nm and 1000-nm PS nanospheres, and Rhodamine 6G (R6G, 99%) were purchased from Sigma Aldrich (Darmstadt, Germany). A 1-wt.% aqueous suspension of 50-nm PS nanospheres was purchased from Phosphorex (Hopkinton, MA, United States). Acetone and absolute ethanol (99.5%, VWR chemicals, Oslo, Norway) were used as received without further purification.

### 2.2 Fabrication of RSN arrays

Glass slides were cleaned with acetone, ethanol and ultrapure water, dried using a gentle stream of nitrogen gas, and then subjected to an oxygen plasma for 1 min (power 100 W, O_2_ flow-rate 50 sccm). A 1-cm diameter ring of polydimethylsiloxane (PDMS) of approximate height 1 mm was deposited on each glass slide, creating a well into which was deposited a 0.5-µL droplet of 500-nm polystyrene nanospheres dispersed in a 2:1 mixture of water and ethanol. The droplets inside each PDMS ring were allowed to dry under ambient conditions, leaving a close-packed monolayer of nanospheres on the surface of each slide. The nanospheres were then treated with an oxygen plasma (power 100 W, O_2_ flow rate 50 sccm) for 10 min, causing them to shrink to a diameter of ∼380 nm while remaining in position. A 5-nm titanium adhesion layer followed by a 45-nm gold film (M1) was deposited by e-beam deposition onto each substrate. (For simplicity we refer to the bilayer Ti (5 nm)/Au (45 nm) films as “gold-films” in the following text). Next, the PS nanospheres were removed by applying 3M scotch tape and then peeling it away, leaving behind a thin gold film patterned with a hexagonal array of nanoholes on each substrate.

The substrates were then cleaned by oxygen plasma for 3 minutes, before being soaked in a 2-mmol ethanolic solution of ODT for 2 h, causing ODT molecules to attach to the upper surface and vertical side walls of M1. The ODT/metal coated substrates were thermally annealed at 80 °C for 30 s in air, before rinsing lightly in ethanol to remove unbound/residual ODT molecules. A 5-nm titanium layer followed by a 45-nm layer of gold (M2) was deposited over the entire area of each substrate. The adhesion between the second metal layer and the outwardly facing alkyl tails of the ODT molecules is very weak, and the parts of M2 that lay directly above the ODT molecules could therefore be removed by gently applying adhesive tape and then peeling it away, leaving M1 and M2 sitting side by side on the substrate separated by the SAM molecules. The SAM molecules were removed by subjecting the coated substrate to an oxygen plasma for 5 min (power 100 W, O_2_ (g) flow-rate 50 sccm), yielding a thin gold film patterned with a hexagonal array of air-filled, ring-shaped nanogaps of approximate diameter 380 nm and approximate width 20 nm (Beesley et al., 2014; Luo et al., 2019).

### 2.3 Preparation of tap-water samples and wash-water samples from an infant feeding bottle

Tap water was collected directly from a drinking-water tap fed by polypropylene pipework. Vacuum filtration (Buchner filter) was used to remove large particulates greater than around 100 nm by successively filtering through glass-fibre filter membranes with pore diameters of 0.75 μm (Whatman, United Kingdom) and 0.1 μm (Xingyacailiao, China). A glass pipette was used to transfer about 1 mL of filtered tap water to an amber-coloured glass sample bottle for further analysis.

A 300-mL PET infant feeding bottle was sterilized in ultrapure water using a steam-based sterilizer for 10 min at 100°C, rinsed with 100 mL of ultrapure water, and then shaken for 1 min at 180 rpm in an automatic shaker (Heidolph, Schwabach, Germany). Thereafter the wash-water was collected and filtered following the same procedure used for tap water. Glass vials were used during the experiment.

### 2.4 Treatment of glassware

Except for a Millipore Glass Base used for vacuum filtration, all glassware and glass-fibre filter membranes were baked for 4 hours in an oven at 500 °C prior to use. The Millipore glass base was rinsed with 1M hydrochloric acid, 1M potassium hydroxide and dichloromethane, and suction filtration was carried out in a fume-hood. Control experiments were performed using ultrapure water in place of the aqueous MNP samples.

### 2.5 Raman measurements

For R6G dose-response measurements, 0.2-µL droplets containing ethanolic solutions of R6G of varying concentrations in the range 10^–4^ M to 10^–12^ M were drop-cast onto clean RSN arrays, dried under ambient conditions, and then directly analysed by Raman spectroscopy without further treatment.

For polystyrene NP measurements, 0.2-µL aqueous droplets containing 1000-nm and 200-nm PS nanoparticles of concentration 1 wt% and 50-nm PS nanoparticles of varying concentrations from 1 wt% to 0.0001 wt% were drop-cast onto gold RSN arrays and dried under ambient conditions. Likewise, 0.2-µL droplets of filtered tap-water and filtered wash-water from an infant feeding bottle were drop-cast onto RSN arrays and dried under ambient conditions.

Raman spectra were obtained on a Horiba confocal Raman spectrometer, using 1-mW laser excitation at a wavelength of 633 nm. The laser beam was focused onto the sample through a ×50 objective lens, yielding a spot-size of around 1 µm in diameter, and all Raman spectra were acquired using a 10-s acquisition time.

### 2.6 Imaging

SEM images were recorded on an electron microscope (FEI APREO) using an electron-beam voltage of 10 kV and a current of 13 pA.

### 2.7 Nanoparticle tracking analysis

A Nanosight LM10 characterisation system (Malvern Panalytical, England) was used for particle sizing by nanoparticle tracking analysis (NTA). A 0.5-mL analyte solution was introduced into the optical viewing cell, and 60-s videos were recorded for each sample. The videos were processed using NTA image analysis software to obtain plots of particle size distribution (PSD).

## 3 Results and discussion

### 3.1 Fabrication and characterization of RSN arrays

**FIGURE 1:**
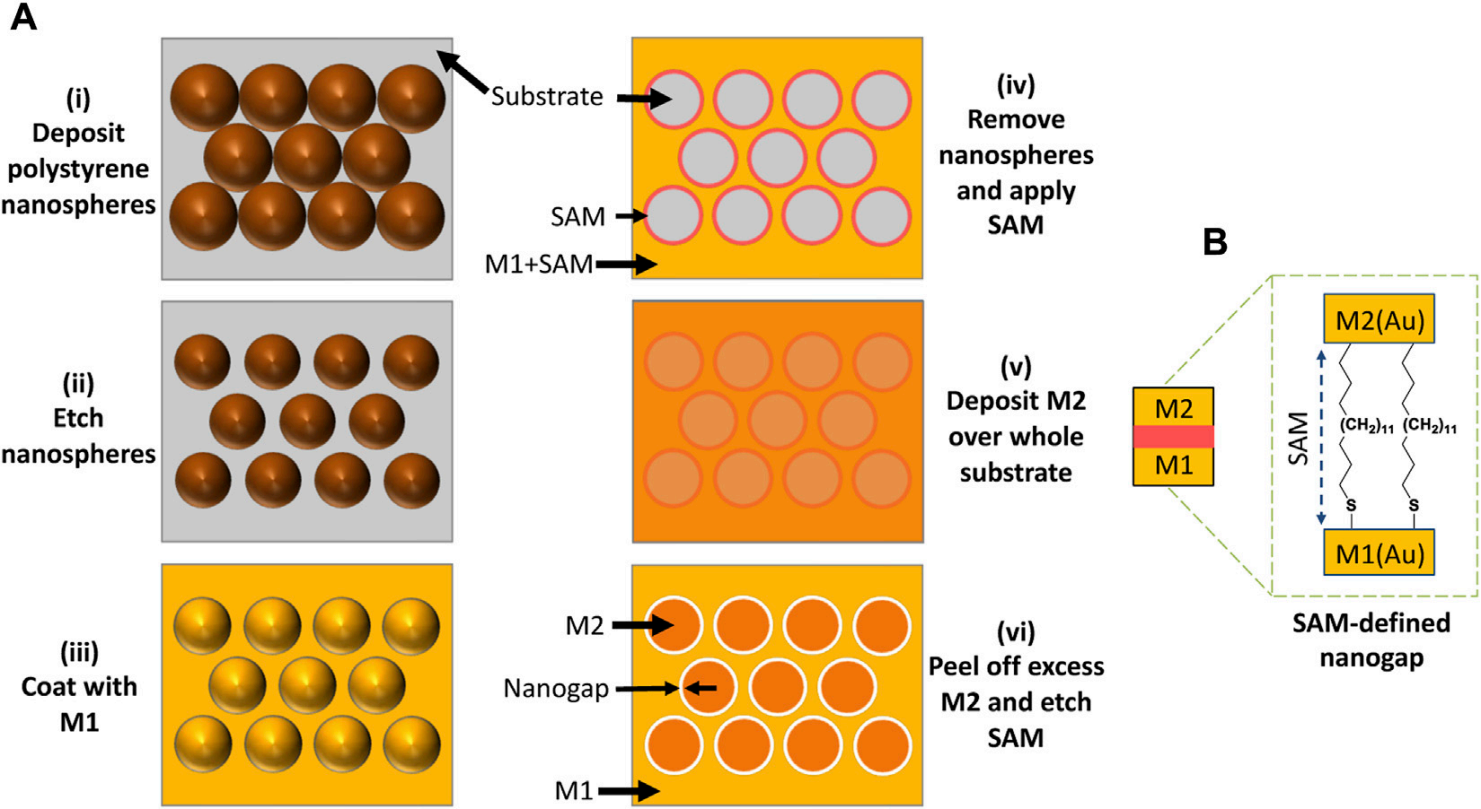
**(A)** Fabrication of nanoring arrays using a combination of nanosphere lithography and adhesion lithography, in which: (i) a monolayer of close-packed polystyrene nanospheres is deposited on a substrate; (ii) the nanospheres are “shrunk” by oxygen plasma treatment, leaving voids between them; (iii) a metal (M1 = Au) is deposited on the substrate through the nanosphere template; (iv) the template is removed and a SAM is applied to M1, leaving a hexagonal array of nanoholes in a SAM-coated Au film; (v) a second metal (M2 = Au) is deposited over the entire substrate; and (vi) excess M2 is removed from the substrate by peeling and the SAM molecules are removed by oxygen plasma treatment, leaving a hexagonal array of ring-shaped nanogaps in gold. **(B)** ODT SAM molecules in the gap-region between M1 and M2 after the peeling step and prior to oxygen plasma treatment.

The procedure used to pattern hexagonal arrays of ring-shaped nanogaps in thin-film gold is depicted in Figure 1. Beesley et al. (2014); Luo et al. (2019) first, a close-packed monolayer of 500-nm diameter PS nanospheres is assembled on a clean glass substrate by drop-casting, see Figures 1A–i. The nanospheres are then reduced in diameter to 380-nm by oxygen plasma etching (Figures 1A–ii). Next, 5 nm of titanium followed by 45 nm of gold (M1) is deposited over the etched nanospheres by e-beam deposition (Figures 1A–iii). The etched nanospheres are removed by tape-stripping, leaving a hexagonal array of ∼380-nm holes in the gold film. A methyl-terminated metallophillic self-assembled monolayer (SAM) of ODT is then conformally attached to the top and vertical sidewall surfaces of M1 by solution-phase deposition (Figures 1A–iv). 5-nm of titanium followed by 45 nm of gold (M2) is then deposited over the entire substrate by e-beam deposition (Figures 1A–v). An adhesive material is applied uniformly to the surface of M2 and peeled away, taking with it those parts of M2 that lie directly above the SAM-coated M1 layer due to poor adhesion between M2 and ODT. After peeling, the two metals M1 and M2 left behind on the glass substrate are arranged side-by-side in a complementary pattern, separated by the SAM molecules. Finally, the SAM molecules are removed by treatment with an oxygen plasma, leaving a hexagonal array of (air-filled) ring-shaped nanogaps of approximate width 10 nm between M1 and M2 (Figures 1A–vi, 1B). (Note, using equal thicknesses for M1 and M2 results in a gap-width that is somewhat larger than the length of the SAM molecules, which is beneficial for the current application as we have previously found that RSNs with gap-widths of around 10 nm yield higher SERS enhancement factors than RSNs with narrower gaps (Luo et al., 2021). For applications requiring a gap-width that is close to the length of the SAM molecules, M2 should be approximately 20 nm thinner than M1 (Luo et al., 2021)) Compared to conventional methods for fabricating nanogap arrays such as e-beam lithography and focused ion-beam milling, the procedure outlined here has the advantage of being fast and scalable to large areas of >50 mm^2^ and involving only a few simple processing steps that can be carried out at room temperature under ambient conditions, using inexpensive equipment.

**FIGURE 2:**
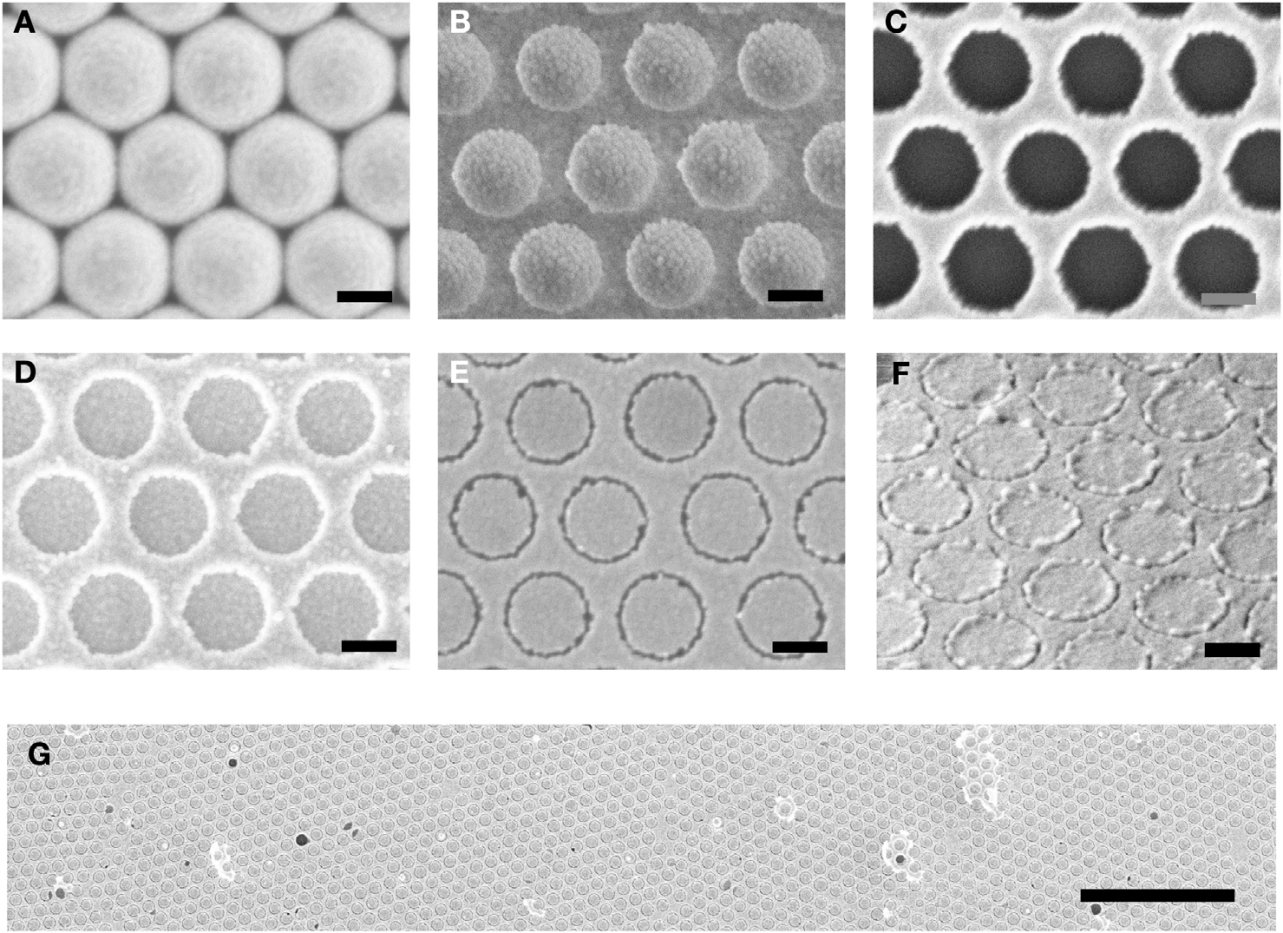
SEM images recorded at key stages in the fabrication procedure. **(A)** close-packed array of 500-nm PS nanospheres on a glass substrate; **(B)** ∼380-nm PS nanospheres on glass after etching with an oxygen plasma; **(C)** hexagonal array of ∼380-nm holes in thin-film gold (M1) obtained after evaporation of gold through the etched PS template and subsequent removal of the PS template by tape stripping; **(D)** layer of gold (M2) on top of hole-patterned gold (M1); **(E)** hexagonal array of ring-shaped nanogaps in gold after removal of excess M2 by peeling and removal of SAM molecules by oxygen plasma treatment; **(F)** tilted image of ring-shaped nanogap array; **(G)** plan-view image of ring-shaped nanogap array at low magnification. Scale-bar in **(A–F)** is 250 nm. Scale-bar in **(G)** is 5 µm.

Figures 2A–E show scanning electron micrographs (SEM) recorded at key stages in the fabrication procedure, using gold for M1 and M2 and octadecanethiol (ODT) as the SAM molecule. Figures 2A, B shows the close-packed array of 500-nm spheres before and after plasma treatment. Figure 2C shows the situation after deposition of the first gold film (M1) and removal of the nanospheres by tape stripping, leaving behind a hexagonal array of ∼380-nm holes in the gold film. Figure 2D shows the situation after deposition of the second gold film (M2) over the entire substrate. Figure 2E shows the final hexagonal RSN array obtained after applying and peeling away an adhesive tape to remove the unwanted parts of M2 and using oxygen plasma treatment to remove the SAM molecules. The dark rings correspond to ring-shaped nanogaps of approximate width 20 nm between M1 and M2. Figures 2F, G show a tilted SEM image and a low-magnification (plan-view) SEM image of the final RSN array. A close-up SEM image and an Atomic Force Microscope (AFM) image of a single ring are shown in Supplementary Figures S1 and S2, indicating a gap-width of around 20 nm. The density of rings in the final array is determined by the diameter of the unetched polystyrene spheres and, for the 500-nm spheres used here, equates to approximately 4.6 million rings per square millimetre.

### 3.2 SERS detection of rhodamine 6G

Under resonant illumination conditions, arrays of metallic nanogaps can give rise to large enhancements in the electromagnetic field due to extreme localization of the incident light in the region of the gaps, which in turn can cause nearby molecules to display a range of surface-enhanced optical properties such as increased absorption, enhanced Raman scattering, second-harmonic generation, and chiroptical behaviour (Chen et al., 2015; Dong et al., 2015; Luo et al., 2021). In a previous paper (Luo et al., 2021), we reported finite element modelling (FEM) of gold RSN arrays with a 3-nm gap width. Under near-resonant excitation by linearly polarised 633-nm plane-wave light, the simulations showed an average intensity-enhancement of at least 335 in the gap region, leading to high SERS enhancement for molecules within the gap. (The calculated intensity enhancement was likely a substantial underestimate of the correct value due to the limited resolution of the spatial grid used).

**FIGURE 3:**
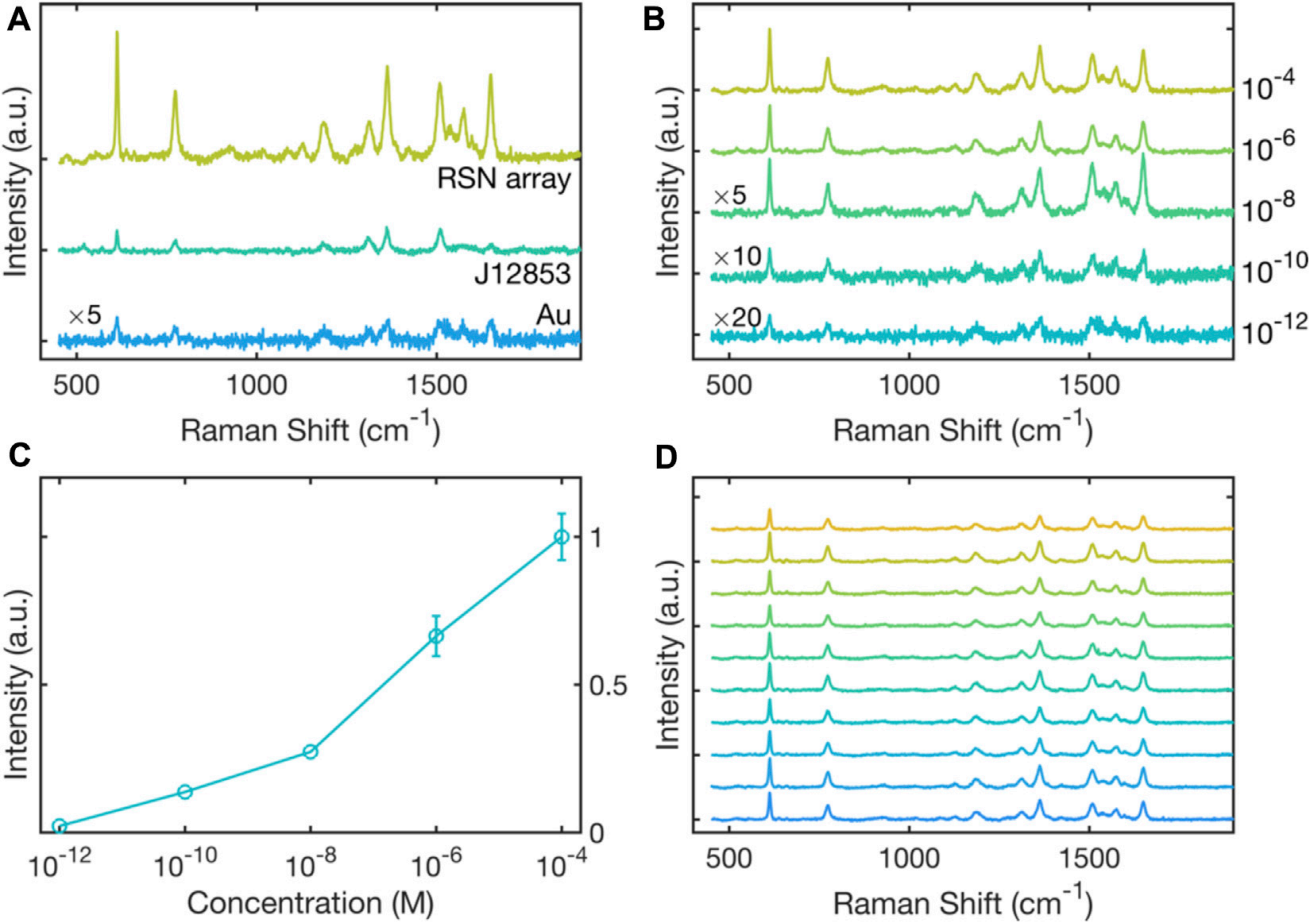
Surface-enhanced Raman scattering of R6G on RSN arrays. **(A)** Raman scattering spectra for 0.2 μL of rhodamine 6G (R6G) drop-cast from a 10^−4^ M solution onto an RSN array fabricated using 500-nm diameter PS nanospheres (etched down to 380 nm). Also shown for comparison is a Raman scattering spectrum for 10^–4^ M R6G drop-cast onto a thin gold film, and 10^–4^ M R6G drop-cast onto a commercial SERS substrate (Hamamatsu J12853) of area 0.07 cm^2^. Spectra were obtained under equivalent conditions with a 633-nm excitation wavelength. The spectrum obtained on thin-film gold has been multiplied by a factor of five for clarity. **(B)** Raman scattering spectra for R6G on RSN arrays drop-cast from R6G dye solutions of varying concentration in the range 10^−4^ M to 10^−12^ M. Spectra were obtained under equivalent conditions using a 633-nm excitation wavelength. The spectra for 10^−8^, 10^–10^ and 10^–12^ M R6G have been multiplied by respective factors of five, ten and twenty for clarity. **(C)** Plot of experimentally determined scattering intensity at 611 cm^−1^ versus dye concentration, extracted from the data in (B). ±1σ error bars have been omitted for concentrations less than or equal to 10^–8^ M as they are smaller than the markers. **(D)** Raman scattering spectra for R6G drop-cast from a 10^−4^ M solution onto an RSN array. Spectra were obtained at ten arbitrary locations on the RSN array under fixed 633-nm illumination.

To test the performance of the RSN arrays as SERS substrates, 0.2 μL of a 10^−4^ M solution of the widely used Raman probe Rhodamine 6G (R6G) was drop-cast onto an RSN array, and a Raman spectrum was recorded at a probe wavelength of 633 nm, see Figure 3A. Also shown in Figure 3A are R6G Raman spectra recorded under equivalent conditions using an unpatterned 50-nm gold film and a commercial SERS substrate with an active area of 0.07 cm^2^ (Hamamatsu, J12853). The signal intensity from the RSN array was much higher than from the thin gold film, consistent with strong enhancement of the electric field inside the ring-shaped nanogaps. The signal intensity from the RSN array was also substantially stronger than that obtained from the commercial substrate.

Next a series of R6G solutions of varying concentration in the range 10^–4^ to 10^–12^ M were drop-cast onto separate RSN arrays, and Raman spectra were recorded under equivalent conditions at a probe wavelength of 633 nm, see Figure 3B. A gradual reduction in the Raman intensity was observed with decreasing dye concentration, but even at 10^–12^ M it was still possible to discern several of the characteristic peaks of R6G. Figure 3C shows the measured signal strength at 611 cm^-1^ versus dye concentration, and indicates an approximately logarithmic relationship between the two. By comparing the 611-cm^-1^ SERS signal from an RSN array coated with a 10^–12^ M R6G solution with the equivalent SERS signal from a thin gold film coated with a 10^–4^ M R6G solution, we derived a SERS enhancement factor of 4.6 × 10^7^ relative to planar gold, see Supplementary Figure S3. Figure 3D shows Raman spectra measured at ten arbitrary spots on the array and indicates a good degree of consistency in the Raman signal from one location to the next, with a coefficient of variation ( $\left.\sigma /\mu \right)$ of around 60%.

Beyond the high enhancement-factor and macroscale uniformity of the SERS signal reported above, we have previously shown that gold RSN arrays fabricated by the general procedure outlined above exhibit excellent environmental stability, with virtually identical SERS signals being obtained from a freshly fabricated array and one that had been stored under ambient conditions for 3 weeks (Luo et al., 2022b). Moreover, we have previously found that the RSN arrays may typically be reused three or more times by using a combination of oxygen plasma treatment and solvent washing to remove deposited material (Luo et al., 2022a). The ability to fabricate the RSN arrays over large areas, combined with their high enhancement factors and excellent environmental stability, make them attractive candidates for many SERS applications.

### 3.3 SERS detection of polystyrene MNPs in stock solutions

Whilst R6G is a dye molecule with strong visible-light absorption, the above results were obtained under non-resonant Raman conditions–i.e., using an excitation wavelength outside the absorbing range of the dye molecule–with the RSN arrays acting as optical antennae to couple light to and from the molecules. The RSN arrays may equally be applied to the SERS detection of analytes with negligible visible-light absorption such as MNPs formed from saturated polymers–the most common form of e-MNPs.

**FIGURE 4:**
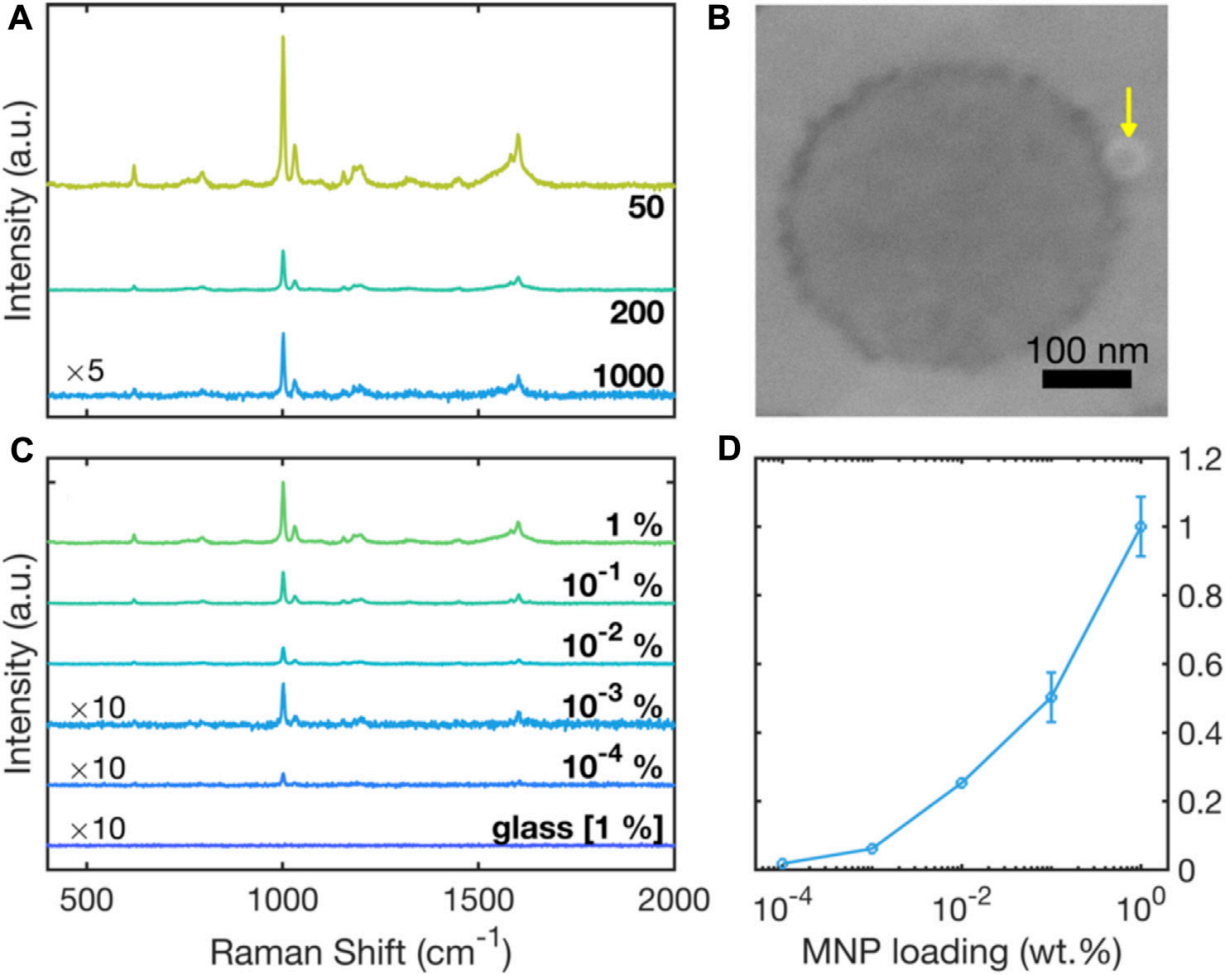
Surface-enhanced Raman scattering spectra due to polystyrene NPs on gold RSN arrays. **(A)** Raman scattering spectra for 0.2 µL of 50-, 200- and 1000-nm diameter polystyrene NPs drop-cast from 1 wt% aqueous solutions. **(B)** SEM image, showing a ∼ 50-nm polystyrene NP (indicated by the yellow arrow) in close proximity to a ring-shaped nanogap. **(C)** Raman scattering spectra for 50-nm polystyrene NPs drop-cast from solutions of different concentrations in the range 1 wt% to 10^–4^ wt%. Also shown for reference is an equivalent Raman scattering spectrum on glass, obtained using a 1 wt% solution of 50-nm polystyrene NPs. The spectra for 10^−3^ wt% and 10^−4^ wt% samples on RSN arrays and the 1 wt% reference sample on glass have been multiplied by a factor of ten for clarity. **(D)** Plot of experimentally determined scattering intensity at 1001 cm^−1^ versus dye concentration, extracted from the data in (C). ±1σ error bars have been omitted for concentrations less than or equal to 10^–2^ wt% as they are smaller than the markers. Data were acquired under equivalent conditions with a 633-nm excitation wavelength.

Figure 4A shows 633-nm Raman spectra for polystyrene nanoparticles of varying diameter, obtained by drop-casting 0.2-µL, 1. wt% aqueous solutions of the PS NPs onto clean RSN arrays. The principal characteristic Raman peaks of polystyrene are evident in all three spectra, with for instance the peaks at 1001 cm^-1^ and at 1032 cm^-1^ corresponding to ring-mode vibrations of C-C and C-H bonds (Xu et al., 2020). A clear increase in the intensity of the Raman signals is evident as the particle size decreases from 1000 nm to 50 nm, consistent with the increasing probability of finding PS chains in close proximity to the ring-shaped nanogaps where they can benefit from the optical antenna effect. (An SEM image showing a 50-nm particle in contact with a ring-shaped nanogap is shown in Figure 4B).

Figure 4C shows 633-nm Raman spectra for 50-nm polystyrene NPs deposited onto the RSN arrays from solutions with polystyrene concentrations between 1 wt% and 10^–4^ wt%. Also shown for reference is an equivalent Raman spectrum for 50-nm polystyrene NPs on a glass substrate, drop-cast from a 1 wt% solution. The spectrum on glass shows no discernible Raman peaks, consistent with extremely weak inelastic scattering of incident light under non-resonant conditions on SERS-inactive substrates. The spectra measured on the RSN arrays by contrast showed Raman peaks at all MNP loadings, with the Raman intensity decreasing gradually as the MNP loading was decreased. Figure 4D shows the 1001 cm^-1^ peak height versus MNP loading, and indicates a super-logarithmic dependence of the scattering signal on the particle concentration.

Even at the lowest MNP loading of 10^–4^ wt%, the main peaks at 1001 cm^-1^ and 1607 cm^-1^ were detectable above the noise-floor of the measurement. 10^–4^ wt% corresponds to a concentration of 1 μg/mL, which is at the lower range of detectable concentrations reported in the literature. Yang et al. (Yang et al., 2022) and Chaisrikhwun et al. (Chaisrikhwun et al., 2023) reported lower detection limits of 0.1 μg/mL, but using much higher sample volumes and more complicated sample handling.

Figs. S4a and S5a show SERS scattering spectra obtained at eight arbitrary locations within a single substrate, using MNP loadings of 10^–3^ wt% and 10^–4^ wt%, respectively. Figs. S4c and S5c show SERS scattering spectra obtained at a single arbitrary location across five different samples, using the same MNP loadings. The data indicate reasonable location-to-location and sample-to-sample reproducibility, with coefficients of variation (σ/μ) of less than 26% at 1001 cm^-1^, see Supplementary Figures S4B, S4D, S5B and S5D. However–due to the highly sublinear dependence of the scattering intensity on the NP concentration (see Figure 4D)—further improvements in the location-to-location and sample-to-sample reproducibility are needed to reliably extract the particle concentration from the intensity of the scattering signal.

### 3.4 SERS detection of polypropylene MNPs in tap water samples

**FIGURE 5:**
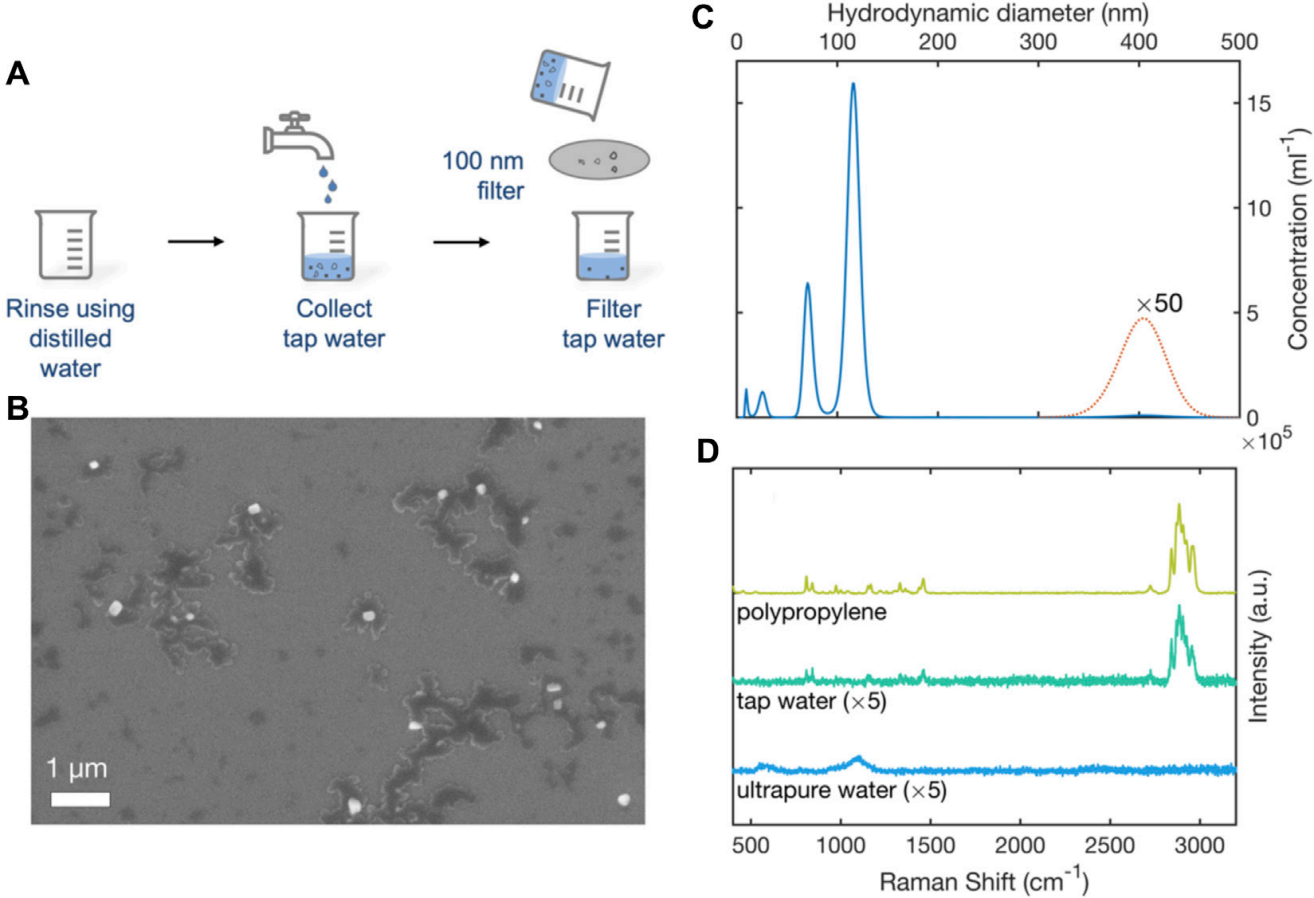
Analysis of e-NPs in tap-water by SEM, NTA and SERS. **(A)** Schematic of experimental procedure used to collect tap-water. Two glass beakers were cleaned with (MNP-free) ultrapure water. A sample of tap-water was collected in one beaker. The tap-water was passed through a 100-nm filter and collected in a second beaker. **(B)** SEM image showing nanoparticulates (white) in the filtered water. Dark regions correspond to residue from dissolved impurities in the water. **(C)** Truncated particle size distribution plot for nanoparticulates in filtered tap-water determined by nanoparticle tracking analysis. **(D)** 633-nm Raman scattering spectra obtained from a polypropylene film, a 0.2-µL sample of filtered tap-water, and a 0.2-µL sample of ultrapure water. Spectra were obtained using RSN arrays under equivalent conditions.

To determine the feasibility of detecting environmental MNPs with the RSN arrays, water was collected from a drinking-water tap fed by polypropylene (PP) pipework–a widely used conduit for potable water (Johnson et al., 2020). The water was passed through a 100-nm glass filter to remove coarse particles, and collected in clean glassware, see Figure 5A. A 0.2-µL sample of the filtered water was deposited onto a clean silicon wafer and imaged by Scanning Electron Microscopy, revealing the presence of particles of typical diameter 100-nm (Figure 5B). Following Lambert and Wagner, Nanoparticle Tracking Analysis was used to obtain a particulate size distribution (Lambert and Wagner, 2016). The extracted PSD plot showed two main peaks at around 71 nm and 116 nm with a much weaker peak at 405 nm. Integrating over the diameter range 0–500 nm, gave a mean particle diameter of 107 nm, a mean particle volume of 1.2 × 10^6^ nm^3^ and a particle concentration of 3.5 × 10^7^ per mL, corresponding to an approximate mass concentration of ∼40 ng/mL (assuming spherical particles and a polypropylene density of 0.9 mg/mL). Truncating the distribution at 200 nm–i.e., omitting 400 nm particles from the calculation–gave a mean particle diameter of 103 nm, a mean particle volume of 7 × 10^5^ nm^3^ and a particle concentration of 3.4 × 10^7^ per mL, corresponding to an approximate mass concentration of ∼20 ng/mL.

0.2-µL samples of tap water were deposited on clean, unused RSN arrays for SERS analysis. For reference purposes, a 1-mm-thick polypropylene film was pressed against an RSN array and tested under identical conditions. Figure 5D shows the resulting surface-enhanced Raman scattering spectra. The close correspondence between the Raman spectrum of the tap-water sample and that of the PP film indicates the presence of PP e-MNPs in the tap-water sample, consistent with the use of polypropylene pipework. The strongest peaks at 1435–1460 cm^-1^ and 2780–2980 cm^-1^ correspond to the -CH_2_ bending and -CH_2_/CH_3_ stretching vibrations of polypropylene, respectively (Käppler et al., 2016; da Silva et al., 2021), suggesting polypropylene was the most abundant nanoplastic in the collected tap water (although other e-MNPs may also be present). The Raman spectrum of the ultrapure water sample by contrast showed none of the features evident in polypropylene reference spectrum.

### 3.5 SERS detection of PET MNPs in the wash-water from a PET-based infant feeding bottle

**FIGURE 6:**
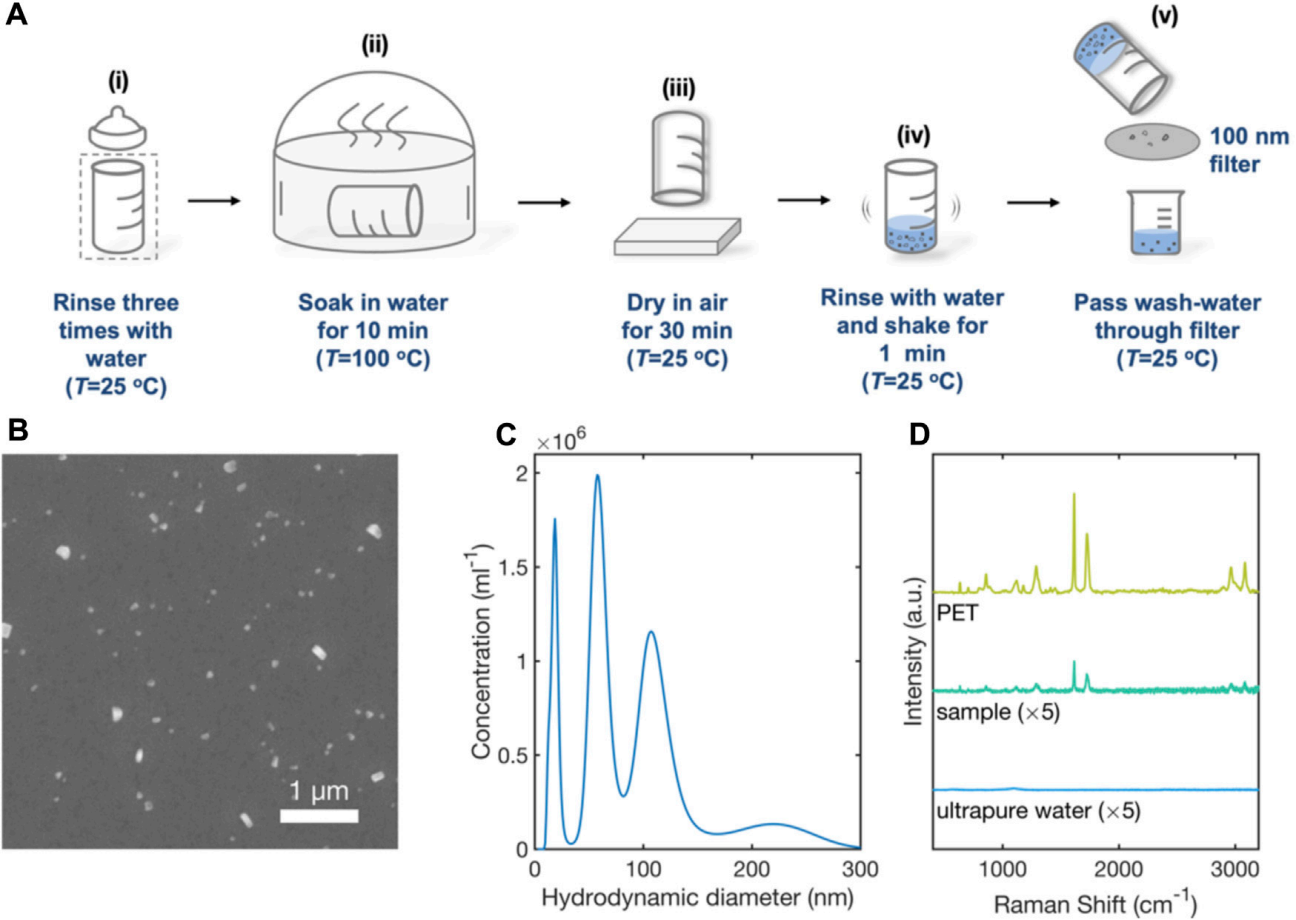
Analysis of e-NPs in the wash-water from a PET-based infant feeding bottle by SEM, NTA and SERS. **(A)** Schematic of experimental procedure used to clean PET bottle and collect wash-water: a PET baby bottle was thoroughly rinsed three times with ultrapure water (i); the bottle was placed in a glass steriliser at 100 °C for 10 minutes (ii), and then left to dry in air at room temperature for 10 minutes (iii). The bottle was filled with 100-mL of ultrapure water and shaken vigorously for 1 minute (iv). The water from the bottle was then passed through a 100-nm filter and collected in a glass beaker (v). **(B)** SEM image showing nanoparticulates (white) in the filtered water. **(C)** Truncated particle size distribution plot for particulates in filtered tap-water determined by nanoparticle tracking analysis. **(D)** 633-nm Raman scattering spectrum obtained from a 0.2-µL sample of wash-water from the baby bottle, a 0.2-µL sample of ultrapure water (control) and a PET film. Spectra were obtained using RSN arrays under equivalent conditions.

Exposure of infants to MNPs from plastic feeding bottles is considered a potential threat to health (Li et al., 2020; Song et al., 2021; Su et al., 2021). To investigate the possible presence of MNPs following a typical bottle-cleaning procedure, an unused PET feeding bottle was rinsed three times with (MNP-free) ultrapure water, placed in a glass sterilizer filled with ultrapure water at 100 °C for a period of 10 minutes, and left to dry in air for 30 minutes at room temperature (see Figure 6A). The clean, dried bottle was then filled with water (mimicking the filling of a bottle with milk after cleaning), and shaken for 1 minute as is typical during the preparation of formula milk. The water from the bottle was then passed through a 100-nm glass-fibre membrane and the filtered water was collected in a glass beaker.

A 0.2-µL sample of the filtered water was deposited on a clean silicon wafer and imaged by Scanning Electron Microscopy, revealing the presence of particles of typical diameter 100-nm (Figure 6B). A separate 0.5-mL sample was analysed by nanoparticle tracking analysis. Integrating over the diameter range 0–300 nm, gave a mean particle diameter of 92 nm, a mean particle volume of 9.9 × 10^5^ nm^3^ and a particle concentration of 11 × 10^7^ per mL, corresponding to an approximate mass concentration of ∼150 ng/mL (assuming spherical particles and a PET density of 1.38 mg/mL).

A 1-mm PET reference film, a 0.2-µL sample of ultrapure water, and a 0.2-µL sample of wash-water from the PET feeding bottle were separately deposited on clean, unused RSN arrays for SERS analysis. Figure 6D shows the resulting surface-enhanced Raman scattering spectra. The Raman spectrum of the wash-water sample corresponds closely to that of the PET film, with strong peaks in the ranges 1435–1460 cm^-1^ and 2780–2980 cm^-1^ that correspond to the -CH2 bending and -CH_2_/CH_3_ stretching vibrations of PET, respectively (Käppler et al., 2016; da Silva et al., 2021). The Raman spectrum for ultrapure water, by contrast, showed no such peaks, indicating the PET NPs were released in the course of the sterilisation process.

The results reported here add to existing evidence in the literature of e-MNP release from plastic feeding bottles, where for instance polypropylene-based infant feeding bottles have previously been shown to release polypropylene microplastics (diameter >800 nm) (Li et al., 2020). In contrast to previous reports that have focused on micro-sized plastics, the results here show nanoplastics are also released during typical handling of feeding bottles, adding to known sources of PET micro/nanoparticle exposure in infants (Zhang et al., 2021).

## 4 Conclusion

Thin gold films patterned with a dense, hexagonal array of ring-shaped nanogaps (RSNs) have been used as substrates for the sensitive detection of MNPs by surface-enhanced Raman spectroscopy (SERS). Compared to previously reported approaches, the approach outlined here has the benefit of using a simple, inexpensive SERS substrate and requiring only small sample volumes and minimal sample preparation. By drop-casting 0.2-μL aqueous test samples onto the SERS substrates, 50-nm polystyrene (PS) nanoparticles could be determined via Raman spectroscopy at concentrations down to 1 μg/mL. The substrates were successfully applied to the detection and identification of ∼100-nm polypropylene e-MNPs in filtered drinking water at an estimated concentration (by NTA) of < 40 ng/mL. They were also applied to the detection of ∼100-nm polyethylene terephthalate (PET) e-MNPs in filtered wash-water from a disinfected PET infant feeding bottle at an estimated concentration (by NTA) of 150 ng/mL.

The principal challenge in using SERS as a quantitative detection method for e-MNP detection is the rather weak, highly sublinear dependence of the scattering intensity on the e-MNP concentration, which demands the use of SERS substrates with high point-to-point and sample-to-sample reproducibility. Future work will therefore focus on the combination of adhesion lithography with alternative lithographic methods that can deliver higher quality patterning of the first metal with a view to further improving the reproducibility of the SERS substrates.

## Data Availability

The data for this study are available on request to the corresponding authors.
