# Supplementary material for: Detection of environmental nanoplastics via surface-enhanced Raman spectroscopy using high-density, ring-shaped nanogap arrays
Source: Front Bioeng Biotechnol. 2023 Oct 24;11:1242797. doi: 10.3389/fbioe.2023.1242797 (PMC10628472; doi:10.3389/fbioe.2023.1242797)
Supplement: Supplementary file 1 [file DataSheet1.pdf]

Detection of environmental nanoplastics via surface-enhanced Raman spectroscopy using high-density, ring-shaped nanogap arrays

Sihai Luo, Junjie Zhang, John C. de Mello

Supporting Information

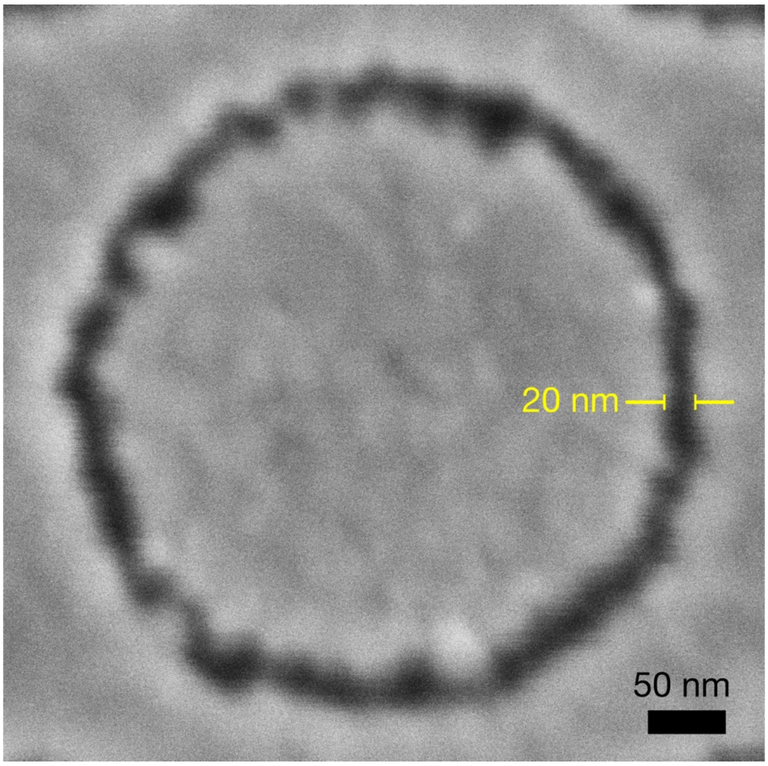

Fig. S1 High resolution SEM image of a gold RSN array, indicating a gap-width of around 20 nm.

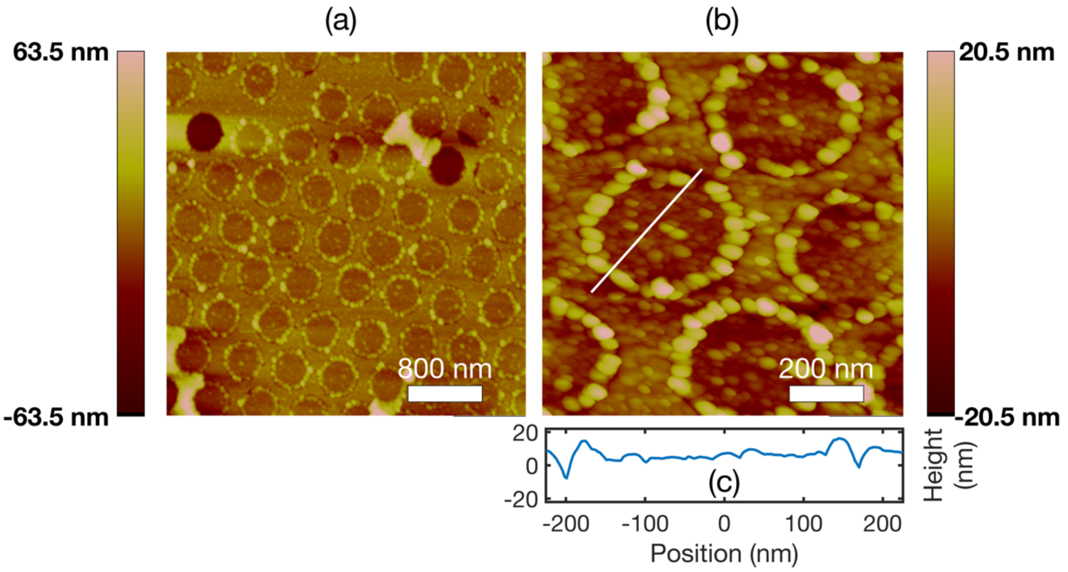

Fig. S2 **(a, b)** Atomic Force Microscope (AFM) images of a gold RSN array at low (a) and high (b) resolution, obtained in tapping mode. **(c)** Line profile along the diagonal white line in (b), with the dips at around -200 nm and +170 nm indicating a gap-width of around 20 nm.

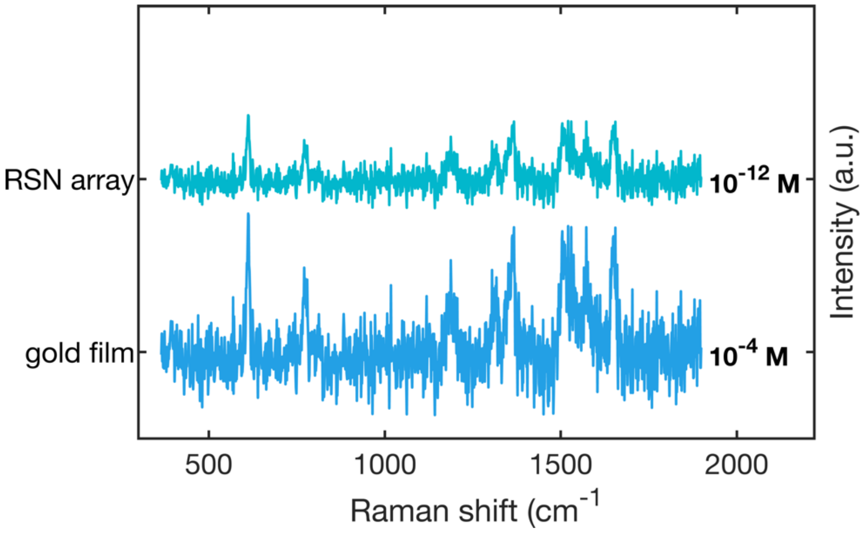

Fig. S3 **Determination of the Raman scattering enhancement factor for R6G on an RSN array relative to R6G on a thin gold film.** The upper trace shows a Raman scattering spectrum for Rhodamine 6G drop-cast from a 10^{-12} M solution onto an RSN array with a pitch of ~500 nm and a ring-diameter of ~380 nm, fabricated using octadecanethiol (ODT) as a spacer. The lower plot shows a Raman scattering spectrum for Rhodamine 6G drop-cast from a 10^{-4} M solution onto a 50-nm gold film. 10^{-12} M and 10^{-4} M represent the respective Raman scattering detection limits for the RSN array and the gold film under the selected acquisition conditions. Spectra were obtained using identical acquisition parameters, see Experimental section. Following Ref. S1, the analytical enhancement factor γ at 611 cm^{-1} was determined using:

$$\gamma = \frac{I_{\mathrm{RSN}}(611\ \mathrm{cm}^{-1})/10^{-12}}{I_{\mathrm{Au}}(611\ \mathrm{cm}^{-1})/10^{-4}} = 4.6 \times 10^{7}.$$

[S1] E. C. Le Ru, E. Blackie, M. Meyer, P. G. Etchegoin, J. Phys. Chem. C. **2007**, 111, 13794.

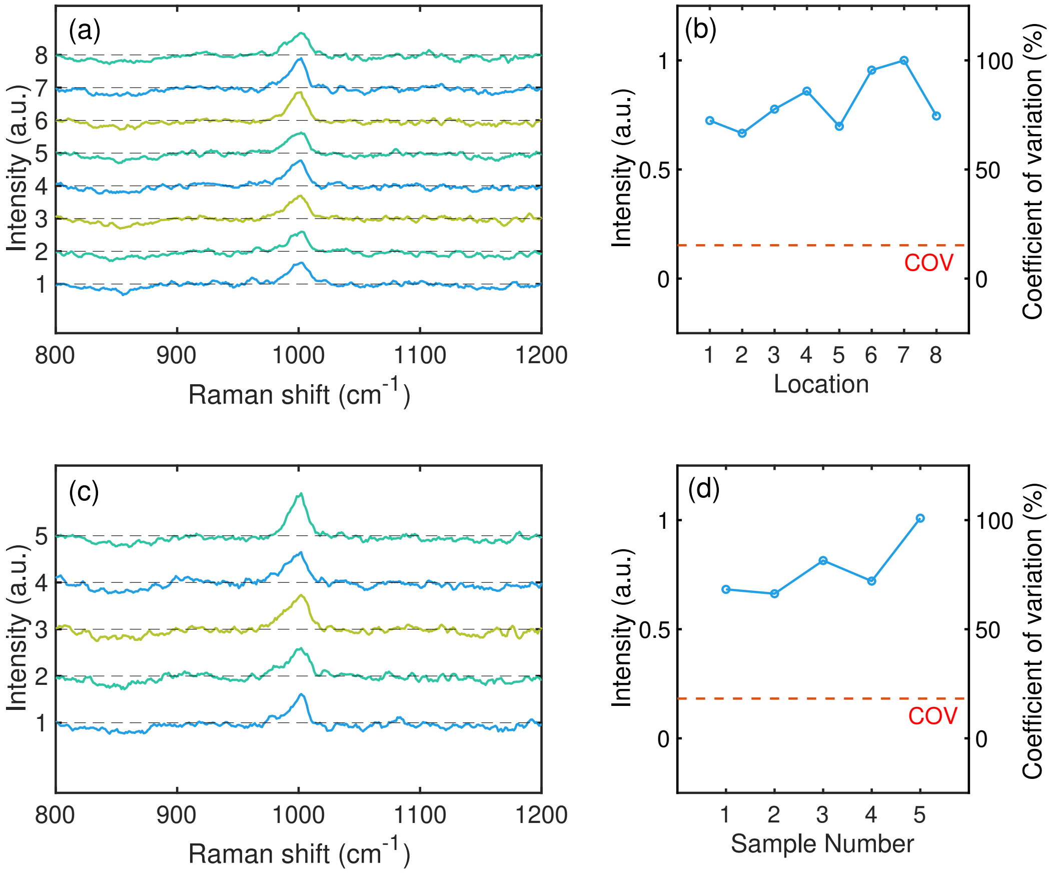

Fig. S4 **Surface-enhanced Raman scattering spectra due to polystyrene NPs drop-cast onto RSN arrays from a 10^{-3} wt.% aqueous dispersion.** SERS spectra obtained at eight arbitrary points on a single substrate **(a)** and associated peak signal intensities at 1001 cm^{-1} **(b)**. SERS spectra obtained at a single arbitrary point on five separate substrates **(c)** and associated peak signal intensities at 1001 cm^{-1} **(d)**. Red dotted lines in (b) and (d) denote the coefficient of variation for the measured signal intensities at 1001 cm^{-1}.

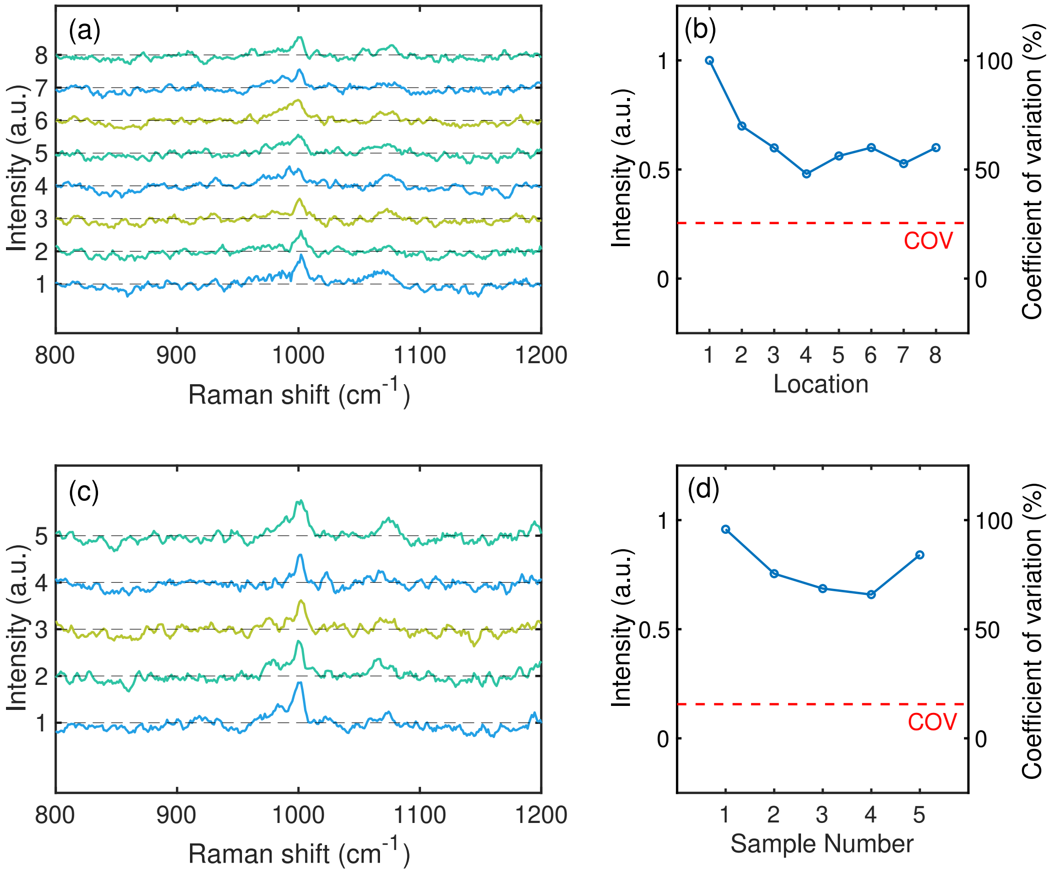

Fig. S5 Surface-enhanced Raman scattering spectra due to polystyrene NPs drop-cast onto RSN arrays from a 10^{-4} wt.% aqueous dispersion. SERS spectra obtained at eight arbitrary points on a single substrate **(a)** and associated peak signal intensities at 1001 cm^{-1} **(b)**. SERS spectra obtained at a single arbitrary point on five separate substrates **(c)** and associated peak signal intensities at 1001 cm^{-1} **(d)**. Red dotted lines in (b) and (d) denote the coefficient of variation for the measured signal intensities at 1001 cm^{-1}.
